# Future Strategies on Glioma Research: From Big Data to the Clinic

**DOI:** 10.1016/j.gpb.2017.07.001

**Published:** 2017-08-07

**Authors:** Hang Cao, Feiyifan Wang, Xue-Jun Li

**Affiliations:** Department of Neurosurgery, Xiangya Hospital, Central South University, Changsha 410008, China

Glioma, as the most common and aggressive malignant central nervous system (CNS) tumor with generally poor prognosis, has been attracting much attention in the last decade [1]. Temozolomide was firstly available in the United States in 1999 as a chemotherapy drug for treating brain cancers and remains as the first-line treatment for glioma. The World Health Organization (WHO) classified glioma into four main grades according to the degree of malignancy in 2007, which were updated in 2016 with the introduction of significant molecular alternations. Also in 2016, the Chinese Glioma Cooperative Group (CGCG) published the first guideline for adult diffuse gliomas [2], representing the only national consensus for the diagnosis and treatment of adult gliomas up till now.

Up till now, hundreds of clinical trials targeted specifically on glioma had been conducted, which unfortunately have not led to a radical improvement in the dismal prognosis of gliomas. Future strategies for glioma research became clear with the establishment of Glioblastoma Multiforme Adaptive Global Innovative Learning Environment (GBM AGILE) and radiomics-associated glioma heterogeneity quantification, as well as development in cancer computational biology and other precision medicine-based GBM studies. The major features of future strategies for glioma research are thus defined to be multi-center involved, big data-based, and computationally-analyzed. Guided by this consensus, the 1^st^ Annual Meeting of Society for Neuro-Oncology of China (SNOChina 2017) was held on March 17–19, 2017 in Beijing, China. The meeting was hosted by the Chinese Medical Doctor Association (CMDA), SNOChina, and Beijing Surgical Institute (BJNI). SNOChina 2017 was initiated by Prof. Tao Jiang, the president of SNOChina and chairman of the subcommittee on brain glioma, CMDA.

SNOChina 2017 was aimed at sharing the clinical management experience, new research progress, and thoughts on the related medical service for glioma. Over 20 Chinese and American leading figures on glioma joined the meeting as invited speakers, together with around 1000 participants domestically and abroad, who are mostly clinicians working on glioma treatment. SNOChina2017 comprised four forums during the 3-day meeting, including (1) Youth Forum, which was focused on the presentation of the latest glioma research progress, mostly about omics data analysis and precision medicine; (2) Academician Forum, which was given by the academicians from Chinese Academy of Sciences (CAS) and Chinese Academy of Engineering (CAE), mainly about the overview of glioma management and research; (3) Master Forum, which was given by the front-line clinicians, mainly about sharing practice skills and future glioma-associated education plan; and (4) International Summit Forum, where overseas specialists reviewed current status for both clinical diagnosis procedures and hospital management of glioma, and provided insightful perspective for the challenges facing and future directions.

This article is intended to introduce the Youth Forum of SNOChina 2017, which could largely represent the active, open-minded attitudes toward glioma, a currently-incurable disease requiring all-time efforts for potential cure.

## Topics

In the afternoon on March 17th, eight speakers reported their recent efforts in glioma research. The forum was started by Prof. Jie Tian, from Institute of Automation, CAS, whose work is focused on multi-modality molecular imaging. Prof. Tian introduced the most pressing challenges for glioma research today and radiomics-based solution strategies. After an overview of radiomics-based studies globally as well as approaches and key technologies employed in such studies, Prof. Tian shared the work of his team applying pre-operation imaging analysis in EGFR mutation prediction for lung cancer and in the curative effect prediction for colorectal cancer. He then introduced the recent project, in collaboration with Beijing Tian Tan Hospital, on epilepsy risk assessment for glioma patient and prediction of non-invasive 1p/19q codeletion [3]. In the end, he shared the efforts of his team in the development of the medical imaging toolkit and its substantial application in over 30 domestic hospitals.

In the following talk, Prof. Guangming Lu from Nanjing General Hospital presented the magnetic resonance imaging (MRI)-based precision diagnosis of glioma. With a brief mention of the clinical and radiological features of glioma, such as the diagnosis criteria, typical subtype distribution, effect of enhancement pattern, and the individualized treatment, Prof. Lu introduced the great value of applying high-resolution MRI, perfusion-weighted imaging, susceptibility imaging, molecular imaging, and Positron emission tomography–MRI (PET/MR) to the diagnosis and differential diagnosis of glioma. Prof. Lu then exemplified the clinical importance of the aforementioned radiological technologies with several case studies. In particular, Prof. Lu shared the efforts of his team in glioblastoma (GBM) outcome analysis by applying multi-modal tissue imaging system. Similarly, Prof. Lu also presented several case studies for radiomics research, focused on the prediction of glioma survival, prediction of mutations in the gene encoding isocitrate dehydrogenase 1 (IDH1), prediction of glioma pseudoprogression, as well as glioma radiation genomics, respectively [4].

Next, Prof. Yu Yao from Huashan Hospital of Fudan University reported the recent progress of his team in glioma immunology after stating the importance of genes encoding IDH1 and telomerase reverse transcriptase (TERT), as well as 1p/19q codeletion in classifying glioma [5]. Afterward, Prof. Yuchen Jiao from Cancer Institute & Hospital, Chinese Academy of Medical Sciences (CAMS & PUMC) focused his talk on glioma genomics and molecular classification. Where is the next biomarker that can substitute IDH1 for prognosis? Prof. Jiao introduced the pancreatic neuroendocrine tumor (PanNET) cancer genome atlas [6] and proposed that given their prognosis power in PanNET, mutations in the gene encoding alpha thalassemia/mental retardation syndrome X-linked/death-domain associated protein (ATRX/DAXX) may play an equally important role in glioma pathogenesis. He then shared several case studies on *ATRX/DAXX* mutations in glioma molecular classification and subsequent treatment.

Prof. Patrick Wen from Harvard University Medical School (HMS) then switched the gear and shared his experience in manuscript preparation. As the editor-in-chief of Neuro-oncology, Prof. Wen firstly introduced the journal, including its submission policy and possible reasons for rejection. As an example, Prof. Wen introduced the properties of the traditional U87 and C6 cell lines. These cell lines are not infiltrating and could not reproduce the human genotype or appropriately predict benefit of drug administered *in vivo*. He thus strongly recommended avoiding using these cells for to perform experiments related to human CNS diseases. And he also mentioned that tumor stem cells derived from glioblastomas cultured in the presence of basic fibroblast growth factor (bFGF) and epidermal growth factor (EGF) mirror the phenotype and genotype of primary tumors more closely than cell lines cultured in the presence of serum, which would be a better human glioma cell model.

Prof. Nu Zhang from the First Affiliated Hospital of Sun Yat-sen University presented recent studies from his team on exploring the potential presence of proteins encoded by circular RNAs (circRNAs) and possible roles of such circRNAs in tumorigenesis. He firstly introduced the general workflow for discovery of coding circRNAs and then further reported biological and clinical functional analysis for the identified coding circRNAs, Circ-PINTexon2 and Circ-FBXW7. Establishing procedure for coding circRNA research, from the initial high-throughput screening, subsequent molecular function validation, to the ultimate application in biomarker discovery, animal model establishment and kit development, may facilitate further code circRNA studies in glioma field.

In the next talk, Prof. Xuejun Li from Xiangya Hospital, Central South University focused his talk on the computational biology strategies for glioma and introduced the recent progress in the lab using these strategies with a few examples. These strategies include (1) identifying glioma subgroups based on consensus clustering and hierarchical clustering methods; (2) integrating omics data for annotation of existing glioma subtypes; (3) predicting prognosis based on the existing subgroups and essential gene mutation data; (4) performing whole-genome enrichment analysis for functional alternations, including GO enrichment analysis, sample level enrichment analysis (SLEA), and gene set enrichment analysis (GSEA) [7]; and (5) establishing molecular networks.

In the last, Prof. Xiang Wang from the West China Hospital, Sichuan University, reported his epidemiological and ethnically-different findings on CNS tumor cases in Sichuan province, China. With 35,496 CNS tumor inpatients of 86 centers enrolled during 2008–2013 from over 81 million Sichuan residents, Prof. Wang reported the significantly lower incidence of high-grade gliomas for Tibetans compared with the Han population [8].

## Conclusion

The first annual meeting of SNOChina was a great success. During the 3-day meeting, more than 100 Chinese glioma-associated hospitals and institutes shared their meeting experience in their social networks. Centered on glioma, this meeting has covered both basic research strategies for precision medicine studies and their potential clinical applications. The concept of big data has already been adopted and applied in glioma research. Its clinical application would be the next hot topic for glioma treatment, which could involve pre-operative radiological diagnosis, subsequent subtype-specific treatment, as well as molecular target drugs. Benefitting from the large number of glioma cases and abundant clinical and genetic materials, Chinese glioma hospitals and institutes would play increasingly important roles in multi-center involved, big data-based, and computationally-analyzed glioma studies.
